# Selection and Optimization of a K_0.5_Na_0.5_NbO_3_-Based Material for Environmentally-Friendly Magnetoelectric Composites

**DOI:** 10.3390/ma13030731

**Published:** 2020-02-05

**Authors:** Michel Venet, Washington Santa-Rosa, Paulo Sergio da Silva, Jean-Claude M’Peko, Pablo Ramos, Harvey Amorín, Miguel Algueró

**Affiliations:** 1 Departamento de Física, Universidade Federal de São Carlos. São Carlos, SP 13565-905, Brazil; washington@df.ufscar.br (W.S.-R.); psergio@df.ufscar.br (P.S.d.S.J.); 2 Instituto de Física de São Carlos, Universidade de São Paulo, São Carlos, SP 13560-970, Brazil; peko@ifsc.usp.br; 3 Departamento de Electrónica, Universidad de Alcalá, 28871 Alcalá de Henares, Spain; pablo.ramos@uah.es; 4 Instituto de Ciencia de Materiales de Madrid (ICMM), CSIC. Cantoblanco, 28049 Madrid, Spain; hamorin@icmm.csic.es (H.A.); malguero@icmm.csic.es (M.A.)

**Keywords:** KNN, lead-free ferroelectric, piezoelectric, magnetoelectric composites, layered composites

## Abstract

Li- and Ta-modified K${}_{0.5}$Na${}_{0.5}$NbO${}_{3}$ compounds are among the most promising lead-free ferroelectrics for high-sensitivity piezoelectric ceramic materials, and are potentially capable of replacing Pb(Zr,Ti)O${}_{3}$. They are also being investigated as piezoelectric components in environmentally friendly magnetoelectric composites. However, most suitable modifications for this application have not been identified. We report here a simulation study of how the magnetoelectric voltage responses of layered composite structures based on Li${}_{x}$(K${}_{0.5}$Na${}_{0.5}$)${}_{1-x}$Nb${}_{1-y}$Ta${}_{y}$O${}_{3}$ varies with the chemical composition of the piezoelectric. Instead of relying on material coefficients from the literature, which would have required using different sources, an ad hoc set of materials was prepared. This demanded tailoring preparation by conventional means to obtain dense ceramics while controlling alkali volatilization, perovskite phase and microstructure, as well as characterizing their dielectric, elastic and electromechanical properties. This provided the set of relevant material coefficients as a function of composition, which was used to obtain the magnetoelectric responses of model layered structures including a reference magnetostrictive spinel oxide by simulation. The piezoelectric material leading to the highest magnetoelectric coefficient was identified, and shown to be different to that showing the highest piezoelectric coefficient. This reflects the dependence of the magnetoelectric response on all material coefficients, along with the complex interplay between composition, processing and properties in K${}_{0.5}$Na${}_{0.5}$NbO${}_{3}$-based ceramics.

## 1. Introduction

Finding novel magnetoelectric (ME) materials with room temperature (RT) performance has been the objective of extensive research over the last few decades [1,2,3], because they can change the concept of electrical and magnetic devices, and enable a range of novel related technologies. Examples are transformers, tunnel junctions, sensors, energy harvesters, random access memories, tunable inductors, tunable filters and phase shifters, to mention but a few [3]. Activity is concentrated in two main lines: (1) single-phase multiferroics [4], and (2) composite approaches combining ferroelectric and ferromagnetic phases [5].

Regarding the first case, most promising results have been obtained for BiFeO${}_{3}$ based perovskite solid solutions, for which distinctive, yet low RT magnetoelectric responses have been reported [6,7,8]. However, and in spite of the extensive research, a single-phase multiferroic capable of enabling the anticipated ME technologies has not been developed [9].

Alternatively, one can obtain much larger magnetoelectric responses from the interaction of ferroelectric and ferromagnetic phases in a composite structure [10]. The largest ME coefficients have been reported by strain-mediation, combining high sensitivity piezoelectric and magnetostrictive phases [3,11,12]. Among bulk composite magnetoelectrics, co-fired ceramic layered structures of ferroelectric perovskite and magnetic spinel oxides provide enhanced reliability in applications, and are suitable for miniaturization [13].

The most commonly used perovskite oxide is ferroelectric Pb(Zr,Ti)O${}_{3}$ (PZT), the current-state-of-the-art material for piezoelectric ceramics, or other PbTiO${}_{3}$ based solid solutions [3]. However, currently enforced legal restrictions on the use of toxic and contaminant materials, such as EU-Directive 2002/95/EC (RoHS), its revision EU-Directive 2011/65/EU (RoHS-2) and EU-Regulation 1907/2006/EC (REACH), require the elimination of lead in electronic devices. This has stimulated a global effort to identify lead-free alternatives to PZT. As a result, several promising lead-free materials have been reported [14,15,16,17,18,19]. Among them, K${}_{0.5}$Na${}_{0.5}$NbO${}_{3}$ (KNN)-based materials stand out because of their high Curie temperature and the capability of tailoring properties for different applications by chemical modification [19,20,21,22]. Specifically, Li- and Ta-modified KNN materials (LKNNT) have been shown to present considerably improved piezoelectric properties, related to the chemically-induced shift of the tetragonal to orthorhombic transition of KNN from a temperature above 200 ${}^{{}^{\circ}}$C down to RT. This results in two-phase co-existence and enhanced properties [23,24,25,26,27], similarly to the case of PZT at its morphotropic phase boundary [28]. Nevertheless, KNN-based ceramics are very difficult to obtain because of the volatile nature of the alkaline elements above 1100 ${}^{{}^{\circ}}$C, which often results in compositional deviations, microstructural degradation and formation of secondary phases [29,30,31]. Alternative preparation techniques have been used to address these issues [32,33,34,35], but they generally increase processing costs, and prevent large-scale production.

KNN-based materials have already been investigated as piezoelectric components in magnetoelectric layered composites, and ME coefficients close to those of PZT-containing materials have been obtained [36,37,38]. Compositions with high piezoelectric coefficients were selected in these works. However, other material properties besides electromechanical ones, such as dielectric permittivity and mechanical compliance, must be considered when the maximization of magnetoelectric coefficients is searched [39]. Indeed, approximate analytical expressions indicate that low permittivity and compliance are advantageous for ME composites [40,41].

Although KNN-based materials have been optimized for their use in piezoelectric devices such as actuators, sensors and transducers, no work has addressed the selection and optimization of compositions for their use as piezoelectric component of layered magnetoelectric composites. This was the aim of this work, where the magnetoelectric voltage response of model layered composite structures consisting of Li${}_{x}$(K${}_{0.5}$Na${}_{0.5}$)${}_{1-x}$Nb${}_{1-y}$Ta${}_{y}$O${}_{3}$ and a reference spinel oxide has been obtained by simulation, as a function of the chemical composition of the piezoelectric. Rather than relying on material coefficients from the literature, which would have required using different sources, and thus, putting together ceramic materials with varying microstructures and often inconsistent perovskite phase evolution, an ad hoc set of materials was prepared. This was done by conventional processing, which was tailored to obtain dense ceramics, while controlling alkali volatilization, of a range of LKNNT materials, including reported compositions with the highest piezoelectric activity. Ceramics were characterized, and the full set of relevant material coefficients was obtained as a function of composition, which was used as an input parameter for the simulations. Magnetoelectric voltage coefficients were derived, and the most suitable materials identified, which matched the performance of analogous composites, including commercial PZTs.

## 2. Methods

### 2.1. Preparation and Characterization of the LKNNT Materials

Li- and Ta-modified KNN materials were chosen after the results of Saito et al. [23]. Nine compositions around Li${}_{0.035}$(K${}_{0.5}$Na${}_{0.5}$)${}_{0.965}$Nb${}_{0.80}$Ta${}_{0.20}$O${}_{3}$, which was reported to show the maximal piezoelectric response in [23], were addressed. Materials studied here were Li${}_{\frac{x}{100}}$(K${}_{0.5}$Na${}_{0.5}$)${}_{\left(1-\frac{x}{100}\right)}$Nb${}_{\left(1-\frac{y}{100}\right)}$Ta${}_{\frac{y}{100}}$O${}_{3}$ with *x* = 3.0, 3.5 and 4.0, and *y* = 15, 20 and 25, which were labeled as LxTy: for instance, L3.0T15 stands for composition Li${}_{0.03}$(K${}_{0.5}$Na${}_{0.5}$)${}_{0.97}$Nb${}_{0.85}$Ta${}_{0.15}$O${}_{3}$.

Powders were synthesized by solid state reaction of stoichiometric mixtures of K${}_{2}$CO${}_{3}$ (Aldrich, > 99 % pure), Na${}_{2}$CO${}_{3}$ (Aldrich, > 99 % pure), Li${}_{2}$CO${}_{3}$ (Alfa Aesar, > 99.999 % pure), Nb${}_{2}$O${}_{5}$ (CBMM, > 99.5 % pure) and Ta${}_{2}$O${}_{5}$ (Across, > 99.99 % pure). A two-step calcination process consisting of two successive thermal treatments at 750 ${}^{{}^{\circ}}$C and 800 ${}^{{}^{\circ}}$C for 5 h was used. Intermediate and final ball millings were carried out in isopropyl alcohol with Y${}_{2}$O${}_{3}$-stabilized zirconia grinding media for 24 h. Green pellets were shaped by uniaxial, followed by isostatic pressing and sintered at different temperatures for 2 h with heating and cooling rates of 4 ${}^{{}^{\circ}}$C/min. The temperature range investigated was selected after dilatometry measurements, performed during the heating of green bodies from room temperature up to 1180 ${}^{{}^{\circ}}$C using a Netzsch - DIL 402 PC apparatus.

Phases were controlled by X-ray diffraction (XRD) with a Shimazdu XRD-6000 diffractometer and CuKα radiation. Data were collected at room temperature in the range 20–60${}^{{}^{\circ}}$ (2θ). In the case of ceramics, a thermal treatment was carried out before the XRD characterization at 600 ${}^{{}^{\circ}}$C for 12 h, in order to relax stresses introduced during machining and polishing, and to recover the equilibrium phase and domain configurations. Density was measured by the Archimedes’ method, and relative densities were obtained using crystallographic densities determined from the cell parameters, derived from XRD data. A FEI scanning electron microscope (model Inspect F-50) was used for the characterization of the powder morphology and ceramic microstructure on polished surfaces. Ceramic capacitors were processed for electrical characterizations by painting silver electrodes on opposite faces of the samples and their sintering at 500 ${}^{{}^{\circ}}$C. Dielectric permittivity was measured at 25 ${}^{{}^{\circ}}$C and 1 kHz, by using an IET 7600 plus high-precision LCR meter. Compliances (s${}_{11}$ and s${}_{12}$) and piezoelectric coefficients (d${}_{31}$) were obtained in poled samples with different geometries (bars and discs) through the Gain-Bandwith method [42], by using the same LCR meter. Poling conditions were 4.5 kV/mm for 30 min at 100 ${}^{{}^{\circ}}$C.

### 2.2. Simulation of the ME Response of Model Composite Layered Structures

Three-layer and multi-layer composite structures consisting of the different nine KNN-based compositions and a reference magnetostrictive oxide were simulated. Spinel CoFe${}_{1.75}$Mn${}_{0.25}$O${}_{4}$ (CFM25) was chosen as a nickel-free compound with enhanced effective piezomagnetic coefficients at reduced magnetic bias field, as compared with high magnetostriction CoFe${}_{2}$O${}_{4}$ [43].

The ME voltage response was derived using Comsol Multiphysics (version 4.2a, Comsol Inc., Burlington, MA, USA). A 3-1 configuration was selected, so that the stacking direction, referred to as 3 direction, was assumed as the poling direction of the piezoelectric, and magnetic fields were applied perpendicularly to it (1 direction). Two fields were simultaneously imposed: a static field to magnetize the magnetostrictive layers, and an alternate magnetic field (10 Oe and 50 Hz) to dynamically strain them (the stimulus). The static field was varied in steps between −1 and 1 kOe, and the dynamic voltage generated in the 3 direction was evaluated (the response, resulting from the charge separated in the mechanically coupled piezoelectric layers). Both three- and two-dimensional models (3D and 2D) were tested. Four-node linear piezoelectric tetrahedron type elements were used by Comsol in the 3D case, while three-node linear piezoelectric triangular type elements were used for the 2D model. More details about this method can be found in reference [44].

## 3. Results and Discussion

### 3.1. Preparation and Characterization of the LKNNT Materials

**Figure 1:**
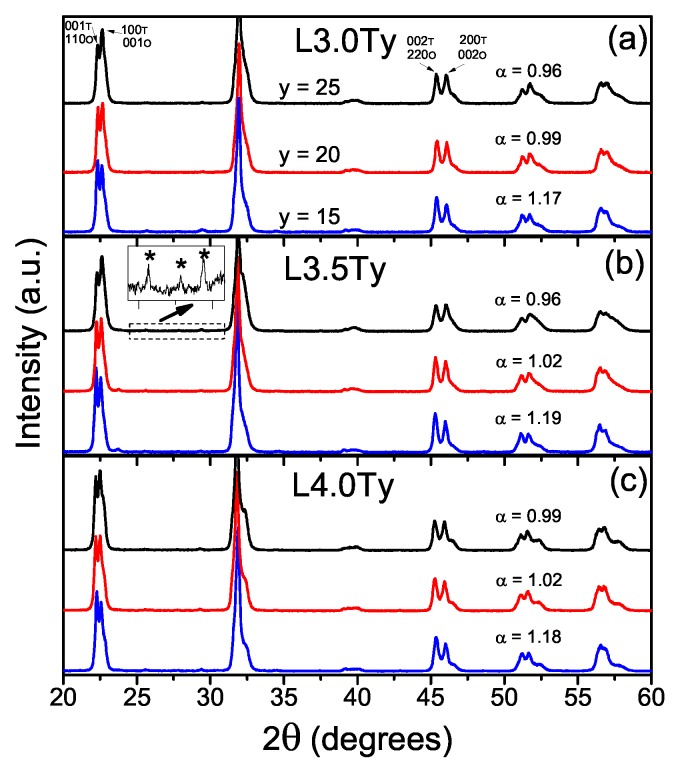
XRD patterns for the Li- and Ta-modified KNN materials (LKNNT) powders with compositions (**a**) L3.0Ty, (**b**) L3.5Ty and (**c**) L4.0Ty. The 001, 100, 002 and 200 peaks of the tetragonal phase were identified in (**a**) with the (T) letter, while the 110, 001, 220 and 002 peaks of the orthorhombic phase were also identified in (**a**) with the (O) letter. The peaks marked with asterisk in (**b**) were identified as belonging to the K${}_{3}$Li${}_{2}$Nb${}_{5}$O${}_{15}$ phase.

The powders were initially characterized by X-ray diffraction (XRD) after the calcination step. Figure 1 shows the XRD patterns for the nine studied LKNNT compositions. All powdered samples were basically perovskite single-phase, but for a small amount of K${}_{3}$Li${}_{2}$Nb${}_{5}$O${}_{15}$ secondary phase with tetragonal tungsten bronze structure, which is commonly found in Li-doped KNN-based materials [45,46]. This secondary phase, present as a very small fraction of the major perovskite phase, is eliminated during the subsequent sintering process.

A large piezoelectric response is associated with the orthorhombic-tetragonal polymorphic phase transition and two-phase coexistence. The presence and percentages of the two perovskite polymorphic phases can be estimated from the XRD patterns with the so-called α parameter, first suggested by Skidmore and Milne [30]. This is based on the relative intensities of the peaks resulting of the splitting of the 100 and 200 peaks of the cubic parent phase. It is given by Equations (1) and (2) for the orthorhombic and tetragonal phases, respectively. Values of α around 1.85 are obtained for materials with orthorhombic symmetry, while values around 0.53 are anticipated for the tetragonal symmetry. Results for the powdered materials are included in Figure 1. Peak areas (integrated intensities) after two-peak deconvolution were used. Note that all materials presented α values between 0.96 and 1.19, which indicates the coexistence of both orthorhombic and tetragonal phases. Additionally, there is a distinctive decrease of the α value when the Ta content increases from 15% to 25%, indicating an increasing tetragonal phase fraction, while variation of Li content between 3% and 4% does not significantly affect the α parameter.

(1) $$\alpha_{\mathrm{ortho}}=\frac{\left(\frac{I_{110}}{I_{001}}+\frac{I_{220}}{I_{002}}\right)}{2}$$

(2) $$\alpha_{\mathrm{tetra}}=\frac{\left(\frac{I_{001}}{I_{100}}+\frac{I_{002}}{I_{200}}\right)}{2}$$

**Figure 2:**
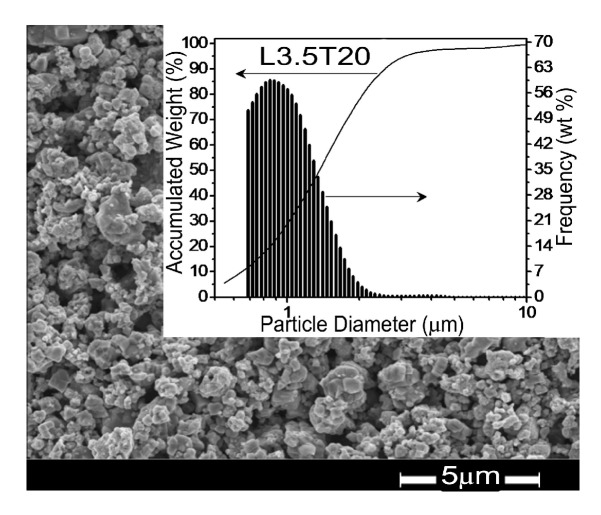
SEM micrograph of the powder with composition L3.5T20. The inset shows the particle size distribution for this powder.

SEM micrographs were taken for all the powders after the final 24 h ball milling, in order to characterize the initial morphology and particle size before the sintering process. No significant differences among powders with different compositions were observed. A representative micrograph corresponding to composition L3.5T20 is shown in Figure 2, along with the particle size distribution obtained with a Sedigraph Micromeritics 5100. Unimodal particle size distributions with average between 0.8 and 1.2 μm resulted with no systematic trend with LKNNT compositions.

**Figure 3:**
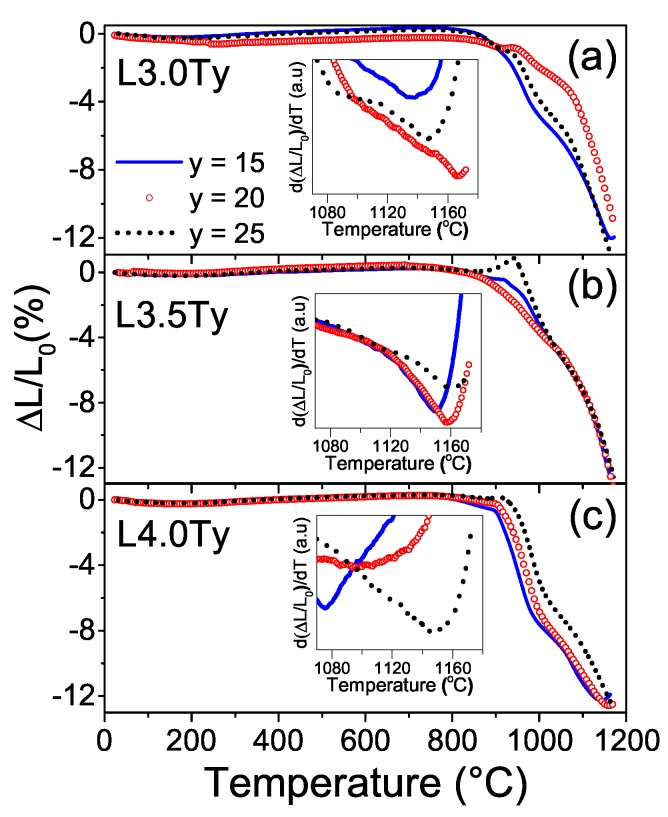
Relative shrinkage and its derivative (inset graphs) as a function of the temperature and Ta content y for the LKNNT powders with compositions (**a**) L3.0Ty, (**b**) L3.5Ty and (**c**) L4.0Ty.

In order to define the sintering conditions to obtain high quality dense ceramics, densification of the green bodies was previously characterized by dilatometry. Shrinkage curves and their derivatives are shown in Figure 3. All compositions showed well defined minima in the derivative curves (indicating maxima in the shrinkage rate), whose position suggests possible temperatures for sintering. Values for the different materials are listed in Table 1. There is a distinctive trend with the Ta content, so that the temperature at which the shrinkage rate is maximal increases with Ta. L3.0T25 deviates from this trend, which could be associated with its average particle size being below the average. No clear trend with Li content was found, though it is worth noting that lowest temperatures took place for L4.0T15 (1075 ${}^{{}^{\circ}}$C) and L4.0T20 (1100 ${}^{{}^{\circ}}$C), while the maximum one was found for L3.0T20 (1166 ${}^{{}^{\circ}}$C). Following these results, L3.5T20 was selected as a representative composition, and conventional sintering experiments were carried out at temperatures varying from 1090 ${}^{{}^{\circ}}$C up to 1160 ${}^{{}^{\circ}}$C, for 2 h.

**Figure 4:**
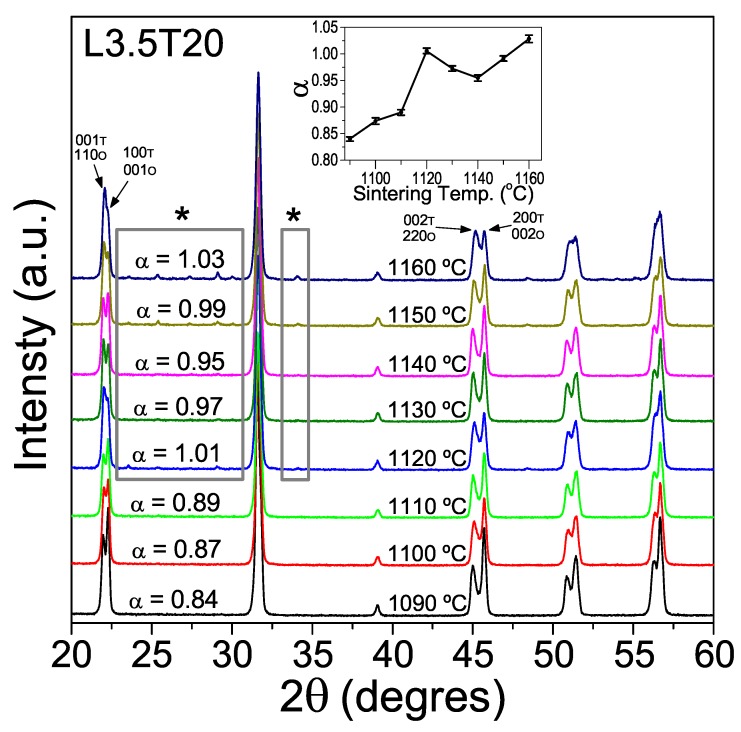
XRD patterns for ceramics with composition L3.5T20, sintered at different temperatures for 2 h. The 001, 100, 002 and 200 peaks of the tetragonal phase were identified with the (T) letter, while the 110, 001, 220 and 002 peaks of the orthorhombic phase were identified in with the (O) letter. Asterisks mark peaks belonging to the secondary phase K${}_{3}$Li${}_{2}$Nb${}_{5}$O${}_{15}$. Inset graph shows the evolution of the α parameter with the sintering temperature.

Figure 4 shows XRD patterns for the ceramics with composition L3.5T20 sintered at increasing temperatures, and the evolution of the α parameter with the sintering temperature. Perovskite single-phase materials were obtained at temperatures below 1120 ${}^{{}^{\circ}}$C, while those sintered between 1120 and 1160 ${}^{{}^{\circ}}$C presented traces of the secondary phase K${}_{3}$Li${}_{2}$Nb${}_{5}$O${}_{15}$. Regarding the α parameter, it remained between 0.84 and 0.89 for ceramics sintered between 1090 and 1110 ${}^{{}^{\circ}}$C, and abruptly increased up to 1.01 for those sintered at 1120 ${}^{{}^{\circ}}$C. Note that the α value for this ceramic is similar to that for the calcined powder of the same composition (1.02), which also had a small amount of the secondary phase K${}_{3}$Li${}_{2}$Nb${}_{5}$O${}_{15}$ (see Figure 1). The abrupt change of the α parameter when the sintering temperature rose up to 1120 ${}^{{}^{\circ}}$C and above, together with the presence of the secondary phase in these cases, could indicate 1120 ${}^{{}^{\circ}}$C to be the temperature at which volatilization of alkaline elements started. This drastically affects the phase-coexistence and relative percentages of phases at RT, which shifts towards the orthorhombic phase.

**Figure 5:**
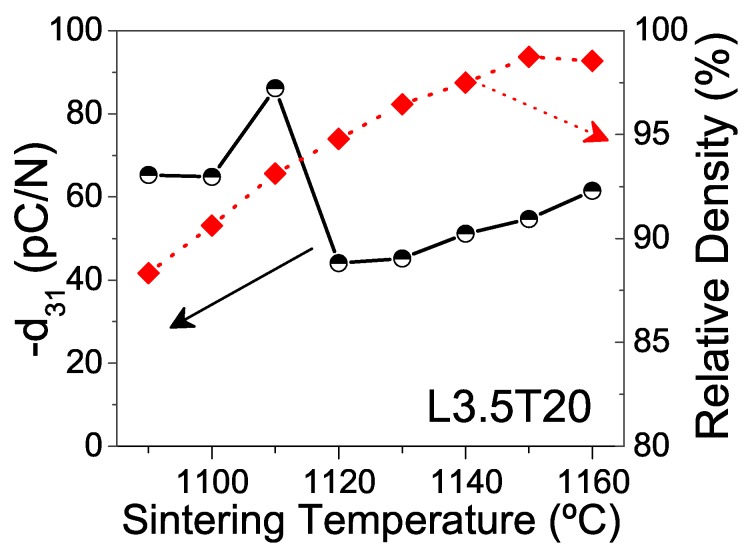
Sintering temperature dependence of the piezoelectric coefficient (d${}_{31}$) and the relative density for ceramics with composition L3.5T20, sintered for 2 h.

This chemical deviation that gives rise to changes in the phase coexistence must also affect properties. Figure 5 shows piezoelectric coefficients d${}_{31}$, functions of the sintering temperature for ceramics with composition L3.5T20. Relative densities are also included. Densification continuously increased with temperature, up to high values ranging from 97% up to 99% for temperatures between 1140 and 1160 ${}^{{}^{\circ}}$C. However, the maximum piezoelectric coefficient d${}_{31}$ was attained in the ceramic sintered at 1110 ${}^{{}^{\circ}}$C, which had a fair relative density of 93%. Indeed, there was a sharp fall in the piezoelectric coefficient d${}_{31}$ when the temperature rose up to 1120 ${}^{{}^{\circ}}$C. This is consistent with the shift in phase coexistence, and thus confirms 1120 ${}^{{}^{\circ}}$C as the temperature, at which volatilization of alkaline elements starts resulting in loss of stoichiometry, formation of secondary phases and degradation of the piezoelectric coefficient.

**Figure 6:**
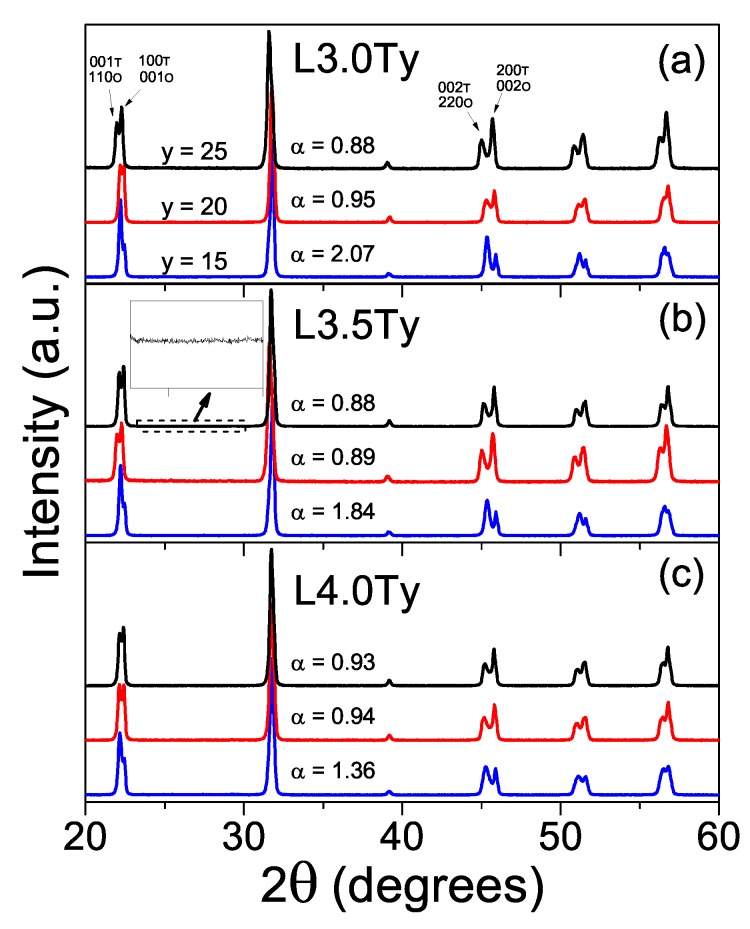
XRD patterns for the LKNNT ceramics, sintered at 1110 ${}^{{}^{\circ}}$C for 2 h, with compositions (**a**) L3.0Ty, (**b**) L3.5Ty and (**c**) L4.0Ty. The 001, 100, 002 and 200 peaks of the tetragonal phase were identified with the (T) letter, while the 110, 001, 220 and 002 peaks of the orthorhombic phase were identified in with the (O) letter.

Considering the results for L3.5T20, 1110 ${}^{{}^{\circ}}$C was chosen as the adequate temperature to process LKNNT ceramics of all compositions. Figure 6 shows the XRD patterns of these ceramics and the calculated α parameter for each composition. Perovskite single-phase materials with no evidence of secondary phases were consistently obtained. Values of α ranged from 2.07 and 1.84 for the L3.0T15 and L3.5T15 compositions, indicating orthorhombic phase (a minor tetragonal phase developed for L4.0T15), down to ∼0.88 for L3.0T25 and L3.5T25 showing mainly tetragonal phase in coexistence with a minor orthorhombic one. As main trend, the tetragonal fraction increased with the Ta content, consistently with previous reports [23].

**Figure 7:**
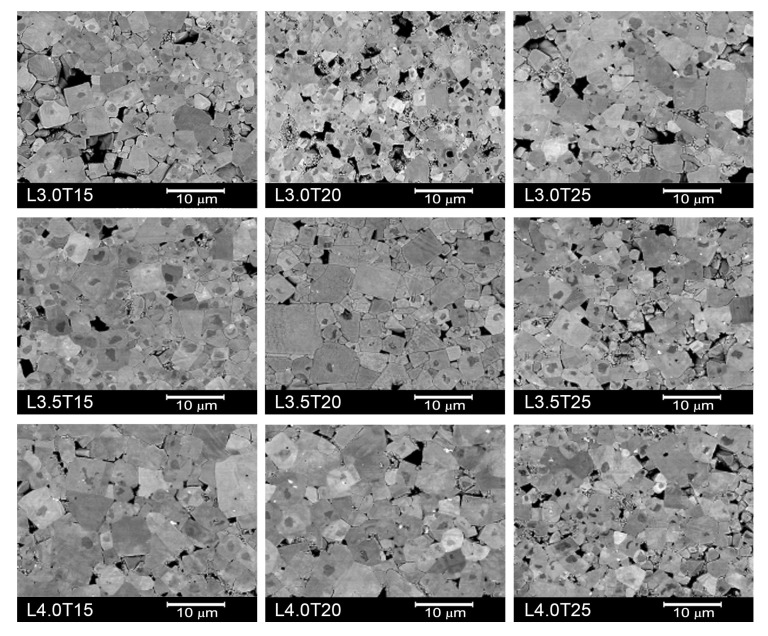
SEM micrographs of the studied LKNNT ceramics, sintered at 1110 ${}^{{}^{\circ}}$C for 2 h.

SEM micrographs for all the LKNNT ceramics sintered at 1110 ${}^{{}^{\circ}}$C are shown in Figure 7. Ceramics L4.0T15 and L4.0T20 showed the lowest porosity fractions, in agreement with their lower temperatures of maximum shrinkage rate, as revealed by the dilatometry results (see Table 1). Note that these two compositions were the only ones for which said temperature was below the onset one for alkali volatilization, and thus, below the temperature used for sintering (1110 ${}^{{}^{\circ}}$C). Additionally, all ceramic materials showed grains with core-shell structure, which is frequently observed in LKNNT materials [31,34,37,47] when conventional solid state synthesis from alkaline carbonates and Ta and Nb oxides is carried out. It has been associated with compositional inhomogeneities, so that Nb- and K-rich cores are surrounded by Ta-rich shells [31].

**Figure 8:**
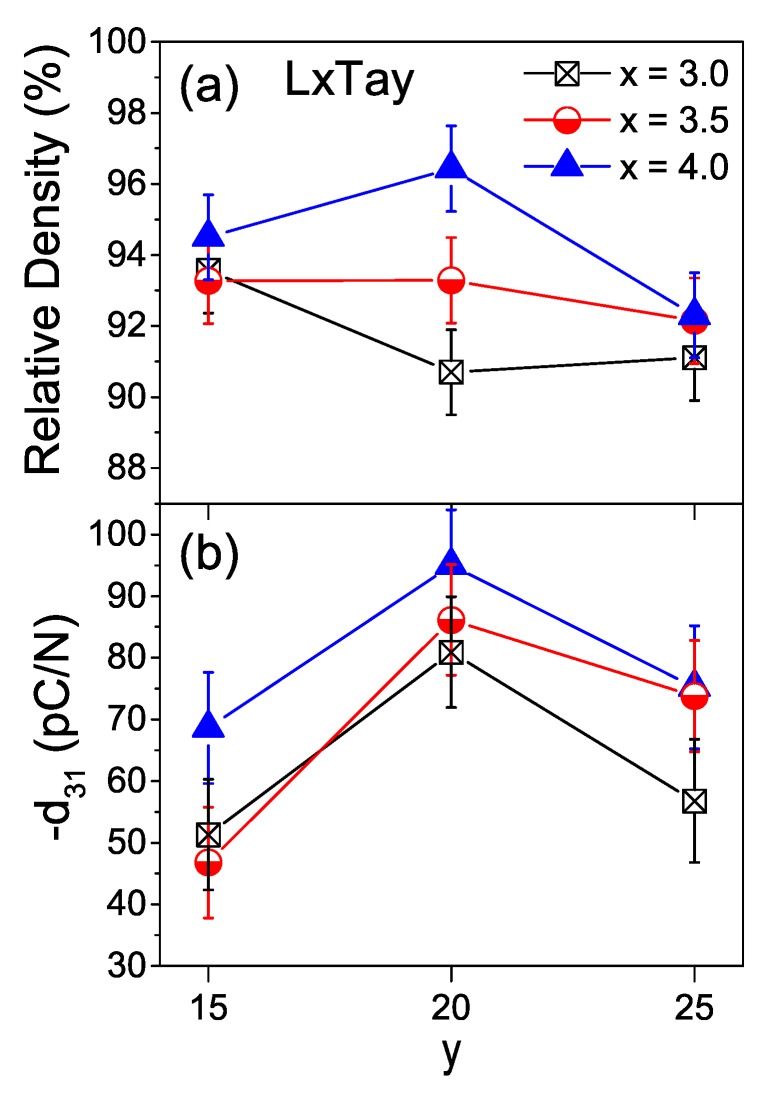
Composition dependence of (**a**) the relative density and (**b**) the piezoelectric coefficient d${}_{31}$ at 25 ${}^{{}^{\circ}}$C for the LKNNT ceramics, sintered at 1110 ${}^{{}^{\circ}}$C for 2 h.

**Figure 9:**
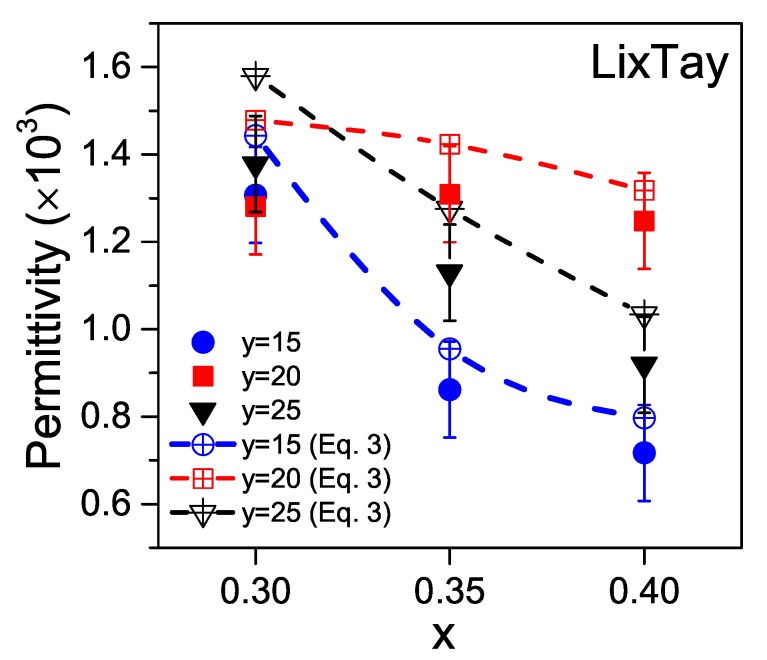
Composition dependence of the dielectric permittivity, at 25 ${}^{{}^{\circ}}$C and 1 kHz, for the LKNNT ceramics, sintered at 1110 ${}^{{}^{\circ}}$C for 2 h. The permittivity values for zero porosity, calculated from Equation (3), were also added and they are represented by open symbols. Dashed lines are guides for the eye.

Figure 8 shows the piezoelectric coefficients d${}_{31}$ for all the LKNNT ceramic materials, along with their relative densities. Error bars here and in Figure 9 and Table 2 correspond to the dispersion of values among five L3.5T20 ceramic samples sintered at the same temperature of 1110 ${}^{{}^{\circ}}$C. Compositions L4.0T15 and L4.0T20 presented the highest relative density with values above 94%, consistent with the SEM and dilatometry results as discussed above. Densifications between 90% and 94% were attained for the remaining materials, whose temperatures of maximum shrinkage rate were above the sintering temperature. Recall that even if one could raise densifications by increasing sintering temperature, this would trigger the volatilization of alkaline elements and degrade piezoelectric properties, as shown for the L3.5T20 composition (see Figure 5).

The d${}_{31}$ piezoelectric coefficients of all LKNNT ceramics were close to those reported for similar compositions [23,48]. For a given Li content, ceramics have a maximum of d${}_{31}$ when the Ta content is 20 mol%, in agreement with the results of Saito and Takao [23]. In addition, compositions L4.0T15 and L4.0T20 had higher piezoelectric coefficients than compositions with lower Li content and equal Ta one. This is most probably related with their higher density. In particular, the d${}_{31}$ coefficient of composition L4.0T20, 95 pC/N, is among the highest values reported for conventionally processed LKNNT ceramics [23,48].

The magnetoelectric responses of composites do= not only depend on the electromechanical coefficients of the piezoelectric layers, but also on their respective dielectric permittivity and elastic constants. Figure 9 shows the compositional dependence of the dielectric permittivity, at 25 ${}^{{}^{\circ}}$C, for the studied LKNNT ceramics. Permittivity values corrected from porosity are also included to separate compositional effects from densification ones. These were calculated with Equation (3) [49],

(3) $$\varepsilon_{p0}≅\frac{\varepsilon (2+V_{p})}{2(1-V_{p})}$$

where $\varepsilon_{p0}$ is the pore free dielectric permittivity, ε the actual permittivity of the ceramic (consisting of pores and material) and $V_{p}$ is the volume fraction of pores (porosity). The trend with Ta content is similar to that shown for the piezoelectric coefficient. Besides, a clear tendency with Li was found, such that dielectric permittivity decreased when Li content increased. The lowest permittivity values were obtained for L3.5T15 and L4.0T15, which might be advantageous for functional voltage responses, such as the magnetoelectric one of a composite including them [39].

Finally, Table 2 contains compliances s${}_{11}$ and s${}_{12}$, piezoelectric coefficients d${}_{31}$ and dielectric permittivities $\varepsilon_{33}$, at 25 ${}^{{}^{\circ}}$C, for all the studied LKNNT compositions. Once again the elastic constants, s${}_{11}$ and s${}_{12}$, and their sum s${}_{11}$ + s${}_{12}$, of ceramics with a given Li content are maximum for a Ta content of 20 mol%. Low s${}_{11}$ + s${}_{12}$ values are desirable for high magnetoelectric voltage response in composites [39], and the lowest values were found for L3.5T15 and L3.0T15.

### 3.2. Simulation of Model Composite Layered Structures Including the LKNNT Materials

Interestingly, compositions of maximum piezoelectric coefficient, minimum permittivity and minimum averaged compliances differed. They were L4.0T20, L4.0T15 and L3.5T15, respectively. In order to identify the optimum composition to be used as piezoelectric component for magnetoelectric layered composites, the material coefficients listed in Table 2 were used as input parameters to simulate, by finite element analysis, the magnetoelectric voltage responses of model composite three-layer and multi-layer structures consisting of the different LKNNT materials and spinel CoFe${}_{1.75}$Mn${}_{0.25}$O${}_{4}$ (CFM25) as a reference magnetostrictive oxide. Input material coefficients for CFM25 were taken from reference [43], where longitudinal and transverse magnetostriction curves were reported, and from reference [39]. They correspond to dense, coarse grained ceramics obtained by conventional processing, and are summarized in Table 3.

**Figure 10:**
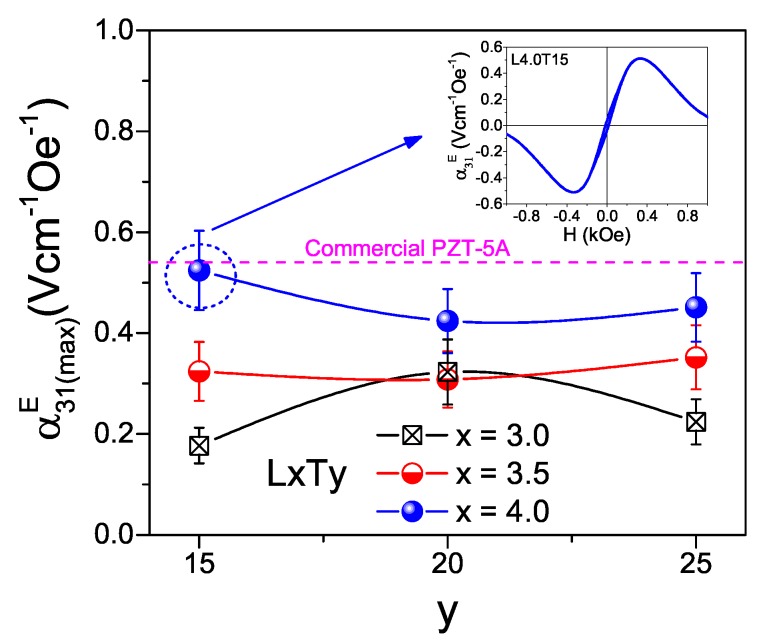
Maximum transverse magnetoelectric (ME) coefficient $\alpha_{31}^{E}$${}_{\left(\max\right)}$, simulated in 2D, as a function of the composition of the piezoelectric phase (LxTay) for three-layer composites CFM25/LxTay/CFM25 and for a geometrically identical three-layer of CFM25/PZT-5A/CFM25 (dashed line). Inset shows $\alpha_{31}^{E}$ as a function of the bias magnetic field (H) for the three-layer CFM25/L4.0Ta15/CFM25.

2D simulations were used to scan all compositions, because they already capture all the physics and allow the optimum composition to be identified, while saving calculation time. Transverse ME coefficients $\alpha_{31}^{E}$${}_{\left(\max\right)}$ were obtained as a function of the static magnetic field. An example is given in the inset of Figure 10. A maximum $\alpha_{31}^{E}$${}_{\left(\max\right)}$ was obtained at a given bias field, which corresponds to the maximum slope in the magnetostriction curve, and is thus characteristic of the magnetic material [43]. Ideal interfaces were assumed; i.e., strain generated in the spinel oxide under the magnetic field is fully transmitted to the perovskite layers. Maximum ME coefficients $\alpha_{31}^{E}$${}_{\left(\max\right)}$ as respective functions of the compositions of the piezoelectric phases (LxTy) are given in Figure 10 for composite CFM25/LxTay/CFM25 structures of 1 mm single-layer thickness (t) and 10 mm length (L). Error bars were obtained by standard error propagation of those associated with the material coefficients given in Table 2 (defining the dispersion of values among five analogously processed piezoelectric ceramics). Composites with piezoelectric phases LxTay having x = 4.0 show the highest $\alpha_{31}^{E}$${}_{\left(\max\right)}$ values. Note that this a consequence of their comparatively low dielectric permittivity. Indeed, the largest ME response occurs for composition L4.0Ta15 that has the lowest permittivity, even if its d${}_{31}$ coefficient is lower than that of most compositions (see Figure 8b). This reflects the complex interplay between composition, processing and properties, along with the role of other properties such as dielectric permittivity and compliance coefficients in maximizing the ME coefficients of composites [39]. In fact, L4.0Ta15 sample also has a relatively low s${}_{11}$ value (see Table 2), which is advantageous for the ME response, as commented on above.

The potential of the selected LKNNT to replace PZT in magnetoelectric layered composites was also evaluated by simulation, comparing the performance of a given model composite structure incorporating the LKNNT material with an analogous one including a commercial PZT instead. Data for PZT-5A from reference [50] were used in this simulation. The obtained magnetoelectric coefficient is included in Figure 10 for comparison. Note that $\alpha_{31}^{E}$${}_{\left(\max\right)}$ for the CFM25/L4.0Ta15/CFM25 layered composite (0.52 Vcm${}^{-1}$Oe${}^{-1}$) is comparable with that for a geometrically identical three-layer structure including Commercial PZT-5A (0.54 Vcm${}^{-1}$Oe${}^{-1}$). This highlights the potential of the L4.0Ta15 composition for its use in environmentally-friendly ME composites with high performance.

**Figure 11:**
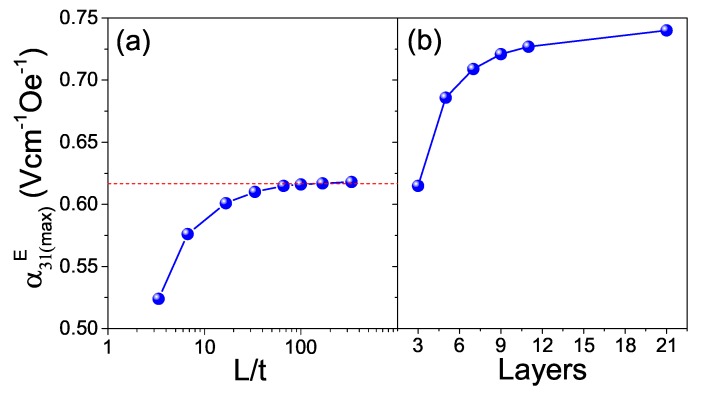
Maximum transverse ME coefficient $\alpha_{31}^{E}$${}_{\left(\max\right)}$, simulated in 2D, as a function of (a) the ratio between length L and thickness t of three-layer composites CFM25/L4.0Ta15/CFM25; and (b) the number of layers for composites with L/t=66, piezoelectric phase L4.0Ta15 and magnetostrictive phase CFM25, which always have the external faces constituted by the magnetostrictive phase.

The magnetoelectric response of a layered composite depends on geometry, an effect that can be easily investigated by simulation. Figure 11a shows how the maximum transverse ME coefficient $\alpha_{31}^{E}$${}_{\left(\max\right)}$ of the model three-layer structures evolves when the ratio between length L and thickness t (L/t) is increased. These simulations were done for CFM25/ L4.0Ta15/CFM25 structures, and a gradual increase of $\alpha_{31}^{E}$${}_{\left(\max\right)}$ with the L/t ratio was found, up to a saturation value of 0.62 Vcm${}^{-1}$Oe${}^{-1}$, which occurs at approximately L/t = 66. Moreover, $\alpha_{31}^{E}$${}_{\left(\max\right)}$ is further enhanced by increasing the number of layers, while maintaining L/t. This is illustrated in Figure 11b, which shows $\alpha_{31}^{E}$${}_{\left(\max\right)}$ of composite multilayer structures of CFM25 and L4.0T15 with L/t = 66 as a function of the number of layers. Indeed $\alpha_{31}^{E}$${}_{\left(\max\right)}$ increases from 0.62 up to 0.74 Vcm${}^{-1}$Oe${}^{-1}$ when the number of layers is increased from 3 to 21. This is partially due to the increment of the piezoelectric volume fraction with the number of layers, from 0.33 to 0.5, at which analytical solutions predict the maximum response [1,41]. Overall, these simulations nicely illustrate how geometry can be tailored to considerably improve the responses of ME composites. Specifically, the magnetoelectric response of composite CFM25/L4.0T15 layered structures can been increased by 42% (from 0.52 to 0.74 Vcm${}^{-1}$Oe${}^{-1}$) by increasing aspect ratio and multilayering.

**Figure 12:**
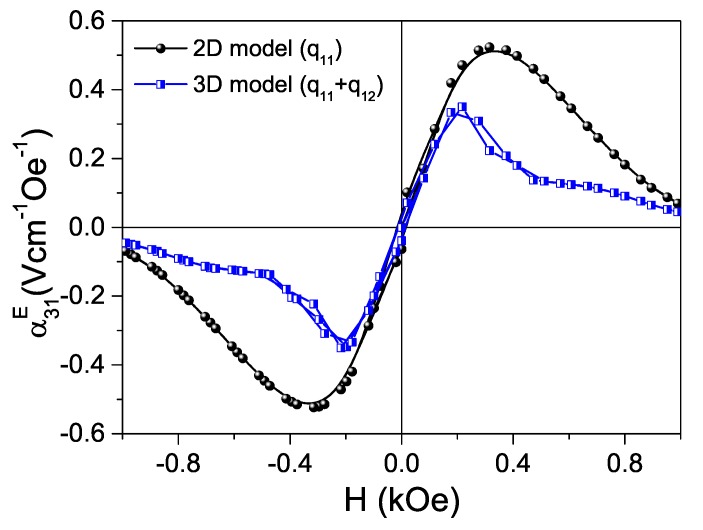
Transverse ME coefficient $\alpha_{31}^{E}$ as a function of the bias magnetic field (H), simulated using 2D and 3D models for the three-layer CFM25/L4.0Ta15/CFM25 with 2R/t (L/t) = 3.3.

2D simulations overestimate magnetoelectric coefficients, because they do not consider transverse magnetostriction. Figure 12 shows transverse ME coefficients $\alpha_{31}^{E}$ as a function of bias magnetic field for composite three-layer discs simulated using a 3D model, compared with the previous 2D simulation. This example corresponds to composite CFM25/L4.0Ta15/CFM25 structures (3D discs or 2D bars) with 2R/t (L/t) = 3.3. As expected, results show a decreased $\alpha_{31}^{E}$${}_{\left(\max\right)}$, together with a displacement of the maximum response towards lower bias magnetic fields, when 3D modeling is used. Note that analogous effects would be obtained for any composition including PZT, so all conclusions reached with 2D simulation hold. Therefore, L4.0T15 can be considered a promising lead-free material for its inclusion in ME layered composites with a performance comparable to those including commercial PZTs.

## 4. Summary and Conclusions

In this work, conventional ceramic processing of Li- and Ta-modified K${}_{0.5}$Na${}_{0.5}$NbO${}_{3}$ was tailored to obtain dense materials with varying chemical compositions around those reported to show high piezoelectric responses, for which material coefficients relevant to their use as piezoelectric phase in magnetoelectric layered composites were obtained. Specifically, a set of dense Li${}_{x}$(K${}_{0.5}$Na${}_{0.5}$)${}_{1-x}$Nb${}_{1-y}$Ta${}_{y}$O${}_{3}$ ceramic materials with x = 0.03, 0.035 and 0.04, and y = 0.15, 0.20 and 0.25, free of secondary phases, with controlled perovskite polymorphic phase coexistence and comparable microstructures, were processed, and their d${}_{31}$ piezoelectric coefficient; $\varepsilon_{33}$ dielectric permittivity; and s${}_{11}$ and s${}_{12}$ mechanical compliances were determined. Characterizations showed that compositions with maximum piezoelectric coefficient and minimum permittivity and compliances differed, which complicated selecting a composition for magnetoelectric layered composites. This was done by simulation with finite element analysis, using the previous material coefficients as input parameters. Model composite three-layer and multilayer structures of the different LKNNT materials and a Mn-modified CoFe${}_{2}$O${}_{4}$ ferrite (CFM25) were modeled, and the magnetoelectric voltage response was derived as a function of the chemical composition of the piezoelectric component. The largest magnetoelectric voltage coefficients were obtained with L4.0Ta15, for which a transverse ME coefficient of 0.52 Vcm${}^{-1}$Oe${}^{-1}$ was obtained, comparable with that for a geometrically identical three-layer structure including a commercial PZT-5A piezoelectric phase instead (0.54 Vcm${}^{-1}$Oe${}^{-1}$). Geometry effects were also simulated, and results illustrated how the magnetoelectric voltage response can be enhanced by increasing the geometric aspect ratio and number of layers. Indeed, a coefficient as high as 0.74 Vcm${}^{-1}$Oe${}^{-1}$ was obtained for composite multilayer structures of L4.0Ta15/CFM25 with an aspect ratio of 66. Results herein presented showed that lead-free piezoelectric Li${}_{0.04}$(K${}_{0.5}$Na${}_{0.5}$)${}_{0.96}$Nb${}_{0.85}$Ta${}_{0.15}$O${}_{3}$ is a promising candidate to be used in environmentally-friendly magnetoelectric composites with high performance.

## Figures and Tables

**Table 1:** Temperature of maximum shrinkage rate (minimum in the derivatives) for each LKNNT composition studied.

	L3.0Ty	L3.5Ty	L4.0Ty
y = 15	1134 ${}^{{}^{\circ}}$C	1156 ${}^{{}^{\circ}}$C	1075 ${}^{{}^{\circ}}$C
y = 20	1166 ${}^{{}^{\circ}}$C	1158 ${}^{{}^{\circ}}$C	1100 ${}^{{}^{\circ}}$C
y = 25	1147 ${}^{{}^{\circ}}$C	1160 ${}^{{}^{\circ}}$C	1150 ${}^{{}^{\circ}}$C

**Table 2:** Properties of the LKNNT ceramics with compositions LxTay at 25 ${}^{{}^{\circ}}$C, including the compliances s${}_{11}$ and s${}_{12}$, their sum s${}_{11}$ + s${}_{12}$, the piezoelectric coefficients d${}_{31}$, the dielectric permittivity $\varepsilon_{33}$ and the relative density $\rho_{\mathrm{rel}}$, in units of 10${}^{-12}$ m${}^{2}$/N, 10${}^{-12}$ C/N and $\varepsilon_{0}$(vacuum permittivity), respectively.

Composition	s${}_{11}\left(\pm 0.8\right)$	s${}_{12}\left(\pm 0.3\right)$	s${}_{11}$ + s${}_{12}\left(\pm 0.9\right)$	d${}_{31}\left(\pm 9.0\right)$	$\varepsilon_{33}\left(\pm 110\right)$	$\rho_{\mathrm{rel}}\left(\pm 1.2\right)$
L3.0Ta15	9.7	−3.1	6.6	−51.3	1307	93.5
L3.0Ta20	14.6	−4.7	10.0	−80.9	1281	90.7
L3.0Ta25	10.6	−3.4	7.2	−56.8	1378	91.1
L3.5Ta15	9.6	−3.1	6.5	−46.8	862	93.3
L3.5Ta20	13.9	−4.5	9.5	−6.1	1309	93.3
L3.5Ta25	11.6	−3.7	7.9	−73.8	1130	92.1
L4.0Ta15	11.4	−3.6	7.7	−68.6	717	94.5
L4.0Ta20	12.0	−3.9	8.2	−95.0	1248	96.4
L4.0Ta25	11.8	−3.8	8.0	−75.2	919	92.3

**Table 3:** Material coefficients for CoFe${}_{1.75}$Mn${}_{0.25}$O${}_{4}$ at 25 ${}^{{}^{\circ}}$C, including the compliances s${}_{11}$ and s${}_{12}$, the maximum piezomagnetic coefficient q${}_{11(max)}$, the magnetic permeability $\mu_{11}$, the dielectric permittivity $\varepsilon_{11}$, the electrical conductivity σ and the density ρ, in units of 10${}^{-12}$ m${}^{2}$/N, 10${}^{-6}$ m/kA, $\mu_{0}$ (vacuum permeability), $\varepsilon_{0}$ (vacuum permittivity), 10${}^{-4}$ S/m and g/cm${}^{3}$, respectively.

s${}_{11}$${}^{†}$	s${}_{12}$${}^{†}$	q${}_{11(\max)}$${}^{‡}$	$\mu_{11}$ ${}^{†}$	$\varepsilon_{11}$ ${}^{†}$	σ ${}^{‡}$	ρ ${}^{‡}$
6.5	-2.4	2.7	2	10	1.2	5.23

${}^{†}$ Values for CFO after Bichurin et al. [39]; ${}^{‡}$ Experimental data from reference [43].
